# The fatigue conundrum

**DOI:** 10.1093/brain/awx153

**Published:** 2017-07-04

**Authors:** Annapoorna Kuppuswamy

**Affiliations:** Sobell Department of Motor Neuroscience, Institute of Neurology, WC1N 3BG, UK

## Abstract

Fatigue is a major affective symptom in neurological disease, but its basis is poorly understood. Kuppuswamy describes the defining features of fatigue and its relationship to perceived effort, before proposing that pathological fatigue is a consequence of impaired sensory attenuation.

## Fatigue: the unexplained phenomenon

‘On an examination of what takes place in fatigue, two series of phenomena demand our attention. The first is the diminution of muscular force. The second is fatigue as a sensation. That is to say, we have a physical fact which can be measured and compared, and a psychic fact which eludes measurement. With regard to the feeling of fatigue, the same thing takes place as happens in the case of every stimulus which acts upon our nerves: we begin to perceive it only when it has attained a certain intensity.’ Angelo Mosso, 1891

One hundred and twenty-five years later, our understanding of fatigue, specifically the sensation of fatigue, has progressed little. Mosso’s description of fatigue is in the context of repetition-induced, reversible, non-pathological, neuromuscular fatigue. However, his observations on the sensation of fatigue are pertinent to our understanding of pathological fatigue. First, the idea of an organic cause for what is essentially a perceptual construct and second, the reference to a feature within the nervous system with attributes of intensity, as the generator to explain the subjective nature of fatigue sensation, are both useful to progress our understanding of the feeling of fatigue.

The aim of this essay is to critically appraise the phenomenology of fatigue and evaluate recent findings to develop plausible mechanistic (functional) hypothesis that may explain pathological fatigue. To this end, I first describe the defining features of pathological fatigue and what sets it apart from other affective symptoms. Second, I elaborate on a potential sensorimotor mechanism, within the framework of active inference, to explain altered effort perception. Third, I make a case for fatigue as a baseline perceptual state and discuss how abnormal perceptual state arises from altered effort perception. Fourth, I discuss fatigue in the context of higher order meta-cognitive functions and finally provide some pointers for future research.

The classic symptoms associated with neurological disorders can be broadly classified into motor, cognitive deficits and affective symptoms. Our understanding of—and ability to quantify and manipulate—motor and cognitive deficits is more developed than our understanding of affective symptoms. However, the resolution of affective symptoms, especially persistent chronic affective symptoms, is a greater priority for many patients. I will assume that affect is fundamentally a product of inference, arising from mental and physical causes. Fatigue is a poorly understood affective symptom, associated with both neuromuscular and cognitive states. Although the notion that physical and mental fatigue are two separate constructs is commonplace in literature, if fatigue in itself is an inference is must be a single construct, irrespective of whether it is based on physical or mental evidence. In light of this, I will use the sensorimotor system to illustrate the basic idea, under the assumption that similar principles can be applied in other (e.g. interoceptive) domains to explain mental fatigue.

Within the gamut of affective symptoms, chronic fatigue is particularly difficult to investigate due to: (i) absence of an obvious trigger; (ii) its subjective nature with no reliable, objective, measurable behavioural surrogate; (iii) its significant overlap with apathy and depression; (iv) the popular belief that fatigue is a secondary symptom, despite evidence to the contrary; (v) the lack of a precise definition; and (vi) confusing terminology. Fatigue and fatigability are used interchangeably, despite recent attempts to clearly distinguish between the two. All of the above are closely linked: absent trigger, subjectivity and overlap result in poor definitions and a confusing terminology leading to skewed popular beliefs. Despite the challenges of investigating fatigue in a systematic and methodical manner, clear ways forward are now emerging.

## Fatigue, apathy and depression

A starting point is to define fatigue, not from an experiential perspective, which, because of its subjective nature, engenders as many definitions as there are respondents, but to define fatigue from a mechanistic perspective. We do not have a good mechanistic understanding of fatigue; however, examining the similarities and differences between fatigue, apathy and depression might throw some light on the nature of fatigue. Behaviourally, all three phenomena are defined by a significant reduction in self-initiated voluntary action. Self-initiated voluntary action calls on two systems; the motivational system to self-initiate, and the executive (sensorimotor) system that delivers voluntary action. It is reasonably well established that the peripheral machinery involved in voluntary action—and to a large extent the central systems directly involved in driving action—are intact, which leaves us with the motivational system. Based on established motivational theories, there are (broadly speaking) two facets to the motivational system; neural processes involved in directional aspects and activational aspects. Directional systems determine if behaviour should be directed towards or away from stimuli; for example, towards food and away from fire and activational systems determine the ‘vigour’ of the behaviour, how quickly to run towards food and away from fire. To summarize, directional systems inform choice and activational systems inform action (Salamone *et al.*, 2016). In the phenomena of interest, fatigue, apathy and depression, a reduction in self-initiated voluntary action can be attributed to dysfunction of either directional or activational systems or both; with subtle differences between the three reflecting a differential dysfunction of the two systems. In apathy and depression, patients normally are not interested in performing actions while in fatigue; despite wanting to act, they feel unable. This subtle distinction could suggest that in apathy and depression, dysfunctional directional systems may play a greater role. Conversely, dysfunctional activational systems may predominate in fatigue. There is no direct evidence to corroborate an activational dysfunction theory of fatigue; however, later I discuss how action cost, a component of the activational system, may be associated with fatigue. A further point of distinction between fatigue, apathy and depression is that fatigue is always self-reported (i.e. a symptom), while apathy and depression can be identified and diagnosed by an external observer (i.e. signs), suggesting fatigue is primarily a perceptual (inference) phenomenon. Based on clinical presentation, subtle distinctions from similar affective symptoms and our understanding of the underlying mechanisms, a plausible definition follows: ‘fatigue is a percept arising primarily from alterations within the activational systems that inform voluntary action’.

## Effort and fatigue

How does a percept, or subjective awareness, of a dysfunction arise? First we must analyse how subjective awareness of a function comes about. For purposes of illustration, let us consider the sensorimotor system. The processes that translate thought into movement are highly automated and for the most part, the agent is unaware of the many processes. However, neural processes that encode information (variables) that could potentially inform explicit choice must have specific properties, which allow the agent to experience or become aware of the ‘information’ encoded. By definition, awareness comes about when a threshold is crossed—or hypothesis is selected; thereby the property of intensity is a prerequisite for any neural process that encodes variables that are experienced by the agent. Within the sensorimotor system, one such variable—which usefully informs both implicit and explicit motor choice (and enters awareness)—is movement/action cost, normally experienced and reported as ‘effort’. ‘Effort’ by definition is an inference or perception; however, there is some confusion in the literature and at times effort is used interchangeably with force, and what is referred to here as effort is normally alluded to as ‘perceived effort’. To avoid any confusion, I use ‘perceived effort’ for the rest of the manuscript.

Perceived effort is a dynamic variable and is heavily influenced by expectations and feedback. Experimental evidence suggests that perceived effort for a given task correlates with pre-movement neural activity and in models of absent afferent feedback, intention to move relates to effort (Lafargue and Franck, 2009). This evidence supports the notion that perceived effort arises from efferent commands, possibly via motor efference copy. However, other studies suggest that perceived effort is significantly altered by manipulating (re)afferent feedback from the muscles. We also know afferent feedback is evaluated centrally in the context of motor intention. Therefore, perceived effort has its origin in intentions (efferent information) that contextualizes feedback (afferent information). In short, ‘perceived effort’ is a perceptual inference that integrates efferent and afferent information.

**Figure 1:**
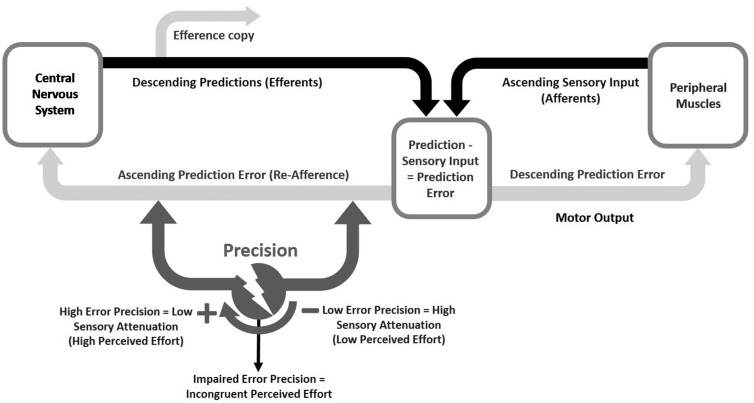
**A schematic representation of how sensory attenuation may underpin perceived effort during movement, using the active inference framework of sensorimotor control.** The descending commands from the brain specify sensory predictions (Efferents) that are compared with the incoming sensory signals (Afferents)—giving rise to sensory prediction errors (Prediction-Sensory Input = Prediction Error). ‘To attend or not to attend’ to the sensory prediction errors, which drive motor output (Descending Prediction Error), depends on the precision the brain affords them (Ascending Prediction Error). When sensory precision is high, prediction errors are heeded to, which I hypothesize gives rise to high perceived effort. On the contrary, when sensory precision is attenuated, afferent sensory errors are ignored resulting in sense of effortlessness. In conditions where sensory attenuation is impaired, incongruent perceived effort may arise leading to perceptual feeling of fatigue.

The active inference framework of sensorimotor control provides a simple framework that integrates efferent and afferent inputs to explain movement initiation and motor control (Brown *et al.*, 2013). Here, I consider how ‘perceived effort’ could be the perceptual consequence of active inference. The active inference framework of sensorimotor control postulates that the (efferent) output from cortico-motor system is in the form of sensory (proprioceptive) predictions and (afferent) input from the somatosensory systems is in the form of sensory (prediction) errors (Fig. 1). On this view, descending predictions of proprioceptive input are fulfilled by classical motor reflexes (or autonomic reflexes in the context of interoceptive predictions). In other words, descending proprioceptive predictions play the role of both motor commands and efference copy, depending on the sensory (proprioceptive or somatosensory) modality predicted. Crucially, to engage reflexes it is necessary to attenuate the precision or gain of afferent or ascending prediction errors that report the fact no movement has been elicited. In short, to perceive the consequences of movement *post hoc*, sensory errors must be heeded to; however, to initiate movement, sensory errors must be ignored. In other words, we have to transiently suspend attention to sensory evidence we are not moving. By altering the precision (or intensity) of sensory prediction errors one can either heed or ignore sensory errors. This function of altering the precision of sensory errors is commonly referred to as ‘sensory attenuation’. Sensory attenuation is elegantly demonstrated in the force matching task. When one is required to match an externally applied force with an internally generated force, one typically overshoots and produces a higher force (Shergill *et al.*, 2003). This overshooting is a result of attenuating the intensity of the sensory consequences of a self-generated motor act, which results in a given force being perceived as less forceful. As a result, when one tries to match the sensation produced by the externally generated force, one overshoots; in other words, one underestimates the force one produces. This is a relatively well studied phenomenon and we now know that sensory attenuation is at its strongest at low force levels and weakest at higher force levels (Walsh *et al.*, 2011).

How might sensory attenuation underpin the experience of movement (i.e. ‘perceived effort’)? If one takes the example of a muscle contraction, under circumstances of normal sensory attenuation, ascending signals (proprioceptive prediction errors) that drive muscle contraction are suppressed (or ignored), which leads to the inference of less or no effort. On the contrary, in the absence of, or when sensory attenuation is poor, the same muscle contraction will be accompanied by ascending proprioceptive prediction errors that can only be explained (by the brain) if the movement requires more effort or work than predicted. It is known that attenuation is stronger in low force muscle contractions than stronger muscle contractions. Similarly, perceived effort has a non-linear relationship with force produced; possibly due to the stronger attenuation at play at low level forces. Although there is no direct evidence, the similarities in ‘force-perceived effort’ and ‘attenuation-muscle contraction’ relationships, strengthens the argument that sensory attenuation underpins perceived effort.

Indirect evidence from disease states further endorses this idea. A classic feature of pathological fatigue is a report of high effort and performing simple activities of daily living. Activities of daily living generally require low levels of muscle force and—under normal circumstances—low level muscle contraction is associated with high sensory attenuation, rendering an ‘effortless’ inference about such activities. However, if sensory attenuation were impaired, daily motor acts would be experienced as effortful—a classic symptom of pathological fatigue. Here, it is useful to remember that pathological fatigue does not correlate with muscle weakness. We know sensory attenuation is incomplete when a system is working at its hardest; for example, when maximal muscle contraction is required. Muscle activity required for simple activities is (near) maximal in a weakened muscle, in the absence of which, one might assume, high perceived effort is a result of impoverished sensory attenuation. Therefore, in chronic pathological fatigue, simple activities feel effortful due to the brains inability to ignore the afferent somatosensory consequences of movement. Prolonged, consistent experience of high perceived effort could therefore eventually lead to the report (or symptom) of fatigue.

## Fatigue, rest and multiple pathologies

We now have a candidate mechanism that potentially explains the predominant feature of chronic pathological fatigue; however, it is as yet unclear how an action-related mechanism explains chronic fatigue at rest. Rest is a word used to describe lack of explicit behaviour; which is not to say the brain is at rest, as spontaneous neuronal firing at ‘rest’ is a well-established fact (Fox and Raichle, 2007). Scientists have only recently started to explore the significance of spontaneous or endogenous neuronal firing. In terms of metabolic cost, spontaneous neuronal firing consumes 20% of body’s energy, while task-related energy consumption accounts for <5% (Fox and Raichle, 2007). In short, ‘resting state’ is a misnomer. What does spontaneous neuronal activity encode? The most popular, mutually non-exclusive theories hypothesize that spontaneous synchronous activity represent a rehearsal of previous task-related use, and the other suggests it rehearses predicted motor scenarios.

If one takes a Bayesian view of the brain, both hypotheses are true—as prior activity informs future predictions; therefore, the rehearsal of completed tasks influences spontaneous neuronal firing, which then informs future actions. In the context of pathological fatigue, a memory of effortful activities (resulting from poor sensory attenuation) influences resting state spontaneous neuronal firing. In a recent resting state functional MRI investigation, pattern recognition techniques have shown that spontaneous fluctuations in resting state neuronal firing relate to individual differences in mood and personality traits, and predict online, self-reported feelings such as sadness. Fatigue, another such feeling, could also be encoded in spontaneous neuronal firing while the brain is at rest. A perceptual state at rest may then arise from spontaneous neuronal activity that is influenced by previous effort-related abnormal (aberrant sensory attenuation) neuronal activity.

Can the above proposed mechanisms of chronic fatigue hold true across several pathological conditions? The mechanisms proposed thus far identify brain-mediated functions that explain an abnormal perception of effort, a salient feature of chronic fatigue. Chronic fatigue is prevalent in a large number of pathological conditions: neurological, autonomic, immunological, hormonal and cardiovascular diseases not to mention a significant side effect of many pharmacological interventions. Here, I propose sensory attenuation as a fundamental mechanism that underpins effort perception, and aberrant sensory attenuation as a disease-independent mediator of fatigue, which in some cases may be the primary driver and in others, a knock-on effect of a more proximal problem. Fundamentally, fatigue can be viewed as the end point manifestation of a cascade of events activated by disease-specific triggers, and—following resolution of the primary trigger—the cascade of events reverse. However, failure to reverse changes in sensory attenuation results in chronicity of the ensuing symptom; namely, fatigue. The point of reversal failure determines if impoverished sensory attenuation is the driver or a mediator of fatigue. A key aspect of these putative failures rests on the similarity between selective attention and sensory attenuation. In active inference, these are both sides of the same coin corresponding to a centrally mediated increase and decrease in precision (i.e. postsynaptic gain of neurons encoding prediction errors at various levels of processing hierarchies). In other words, there is an intimate relationship between attention and attenuation; both of which have to be carefully orchestrated through descending predictions of precision—or top-down gain control. On this view, disruption to higher order cognitive functions such as endogenous attention may contribute to, or be intimately conflated with, the development of fatigue.

## Fatigue and agency

The inability to suppress anticipated neuromuscular sensory consequences of motor commands as a primary cause of pathological fatigue is proposed here for the first time. However, poor sensory attenuation, as demonstrated by a perceptual behavioural task, is an established correlate of disorders of agency (Lafargue and Franck, 2009). How then, can one reconcile the idea of fatigue and disruption of agency as both driven by similar mechanisms? For this, we must examine the common denominator between the two; namely, effort. In fatigue there is a greater sense of effort and in disorders of agency there is a lack (external attribution) of effort. Research is at very early stages and there is little evidence to draw further conclusions; however, one might plausibly speculate on how effort ties together the two seemingly unrelated phenomena. In fatigue (post-stroke), patients often report a loss of control over their body and simple movements require high effort. In disorders of agency, patients attribute control to an external agent or having no control of their actions. One might speculate that different degrees of reported loss of control could be mapped on to different levels of sensory attenuation with: (i) normal sensory attenuation relating to reports of full control over their movements; (ii) in partial sensory attenuation, patients report partial loss of control over their movements, or find it difficult to move their bodies; and (iii) in a state where sensory attenuation fails completely, a total loss of control over their movements is reported, or loss of agency. Moreover, recent evidence suggests effort and sense of agency interact (Demanet *et al.*, 2013). Therefore, fatigue, a perceptual disorder of the sensorimotor system, the system through which the agent fulfils predictions of agency, can be placed within the spectrum of agency-related disorders.

## Concluding remarks and future perspectives

Thus far, I have elaborated a framework within which one can define and understand the symptom of fatigue from a physiological standpoint and place it in the broader context of multiple pathologies. Recent investigations provide indirect evidence for pathological fatigue being a disorder of sensory attenuation in neurological conditions. Low motor cortex resting-state excitability in the stroke affected hemisphere in the fatigue group, despite no difference in sensorimotor impairment and behavioural outcome (Kuppuswamy *et al.*, 2015*b*) raises two questions, the first, ‘does low excitability give rise to fatigue and how?’ Suppression of motor cortex excitability using inhibitory repetitive transcranial magnetic stimulation protocols in healthy volunteers, results in poor sensory attenuation as evidenced by more veridical force matching in the force-matching task, hence low excitability of stroke affected hemisphere may reflect poor sensory attenuation. Second, if sensory attenuation is critical for motor initiation, how might we reconcile the lack of sensorimotor impairment with poor attenuation? In this study (Kuppuswamy *et al.*, 2015*b*) sensorimotor impairment was measured using standard clinical tests that are not sensitive enough to capture subtle alterations in sensorimotor control. However, in a further study we showed that self-selected ballistic movement speeds were indeed compromised in the affected limb of the high fatigue group (Kuppuswamy *et al.*, 2015*a*). Limb heaviness in stroke survivors in relation to fatigue but not muscle weakness, indicates abnormal sensory processing of the affected limb, possibly poor attenuation of sensory afferent information from resting state muscle tone in the affected limb? Attention deficits have been related to post-stroke fatigue (Radman *et al.*, 2012) and attention is inextricably linked to sensory attenuation. Fatigue in multiple sclerosis has been thought of as a disorder of movement preparation as evidenced by altered pre-movement motor cortex excitability and movement preparation includes predictions of sensory consequences—a key feature of sensory attenuation. Interventions targeted at altering motor cortex excitability have thus far shown some positive effects on fatigue; however, direct evidence linking poor sensory attenuation to pathological fatigue is yet to emerge. Future investigations could investigate sensory attenuation in patient cohorts with a wide range of fatigue levels. A simple, established, robust behavioural paradigm that allows one to quantify sensory attenuation is the force matching illusion; see above (Shergill *et al.*, 2003). Furthermore, high frequency neuronal oscillatory activity in motor areas that encode prediction errors and movement parameters both appear to be causally linked to sensory attenuation and neuro-modulatory protocols also support a functional role for motor cortex in sensory attenuation. Therefore, using a combination of behavioural, imaging and neuromodulation techniques, future studies can, in principle, confirm or negate the hypothesis: pathological fatigue is a disorder of sensory attenuation. Interestingly, an explanation based on aberrant sensory attenuation for other affective symptoms, is starting to emerge in computational psychiatry—with the combined use of neuroimaging and dynamic causal modelling to measure sensory attenuation and attention in terms of neuronal gain; i.e. the synaptic efficacy of intrinsic neuronal connections.

Fatigue is a perceptual state that is experienced by all humans, albeit transiently, but when fatigue is non-transient it starts to significantly impact all aspects of the sufferer’s lives. Such fatigue is a hallmark of many pathological conditions and despite more than a hundred years of trying to understand fatigue, there has been very little progress. Here, I highlight the features of fatigue that render it difficult for scientific investigation, propose a unifying definition from a physiological standpoint, elaborate on a disease-independent mechanism that might underlie fatigue, discuss evidence in support of the proposed mechanism and suggest further experiments to verify the hypothesis. If proven to be true, this framework may provide us with the much needed foundation on which to build our understanding of fatigue and, more broadly, a robust link between mind and body.

## Acknowledgements

I would like to extend my sincere thanks to Profs Karl Friston and John Rothwell for the many useful discussions and valuable input to this manuscript. I would also like to thank Dr Sasha Ondobaka and Mr William De Doncker for their feedback on this manuscript.

## Funding

A.K. is funded by the Wellcome Trust 202346/Z/16/Z.
